# Nanocellulose and Nanocarbons Based Hybrid Materials: Synthesis, Characterization and Applications

**DOI:** 10.3390/nano10091800

**Published:** 2020-09-10

**Authors:** Djalal Trache, Vijay Kumar Thakur

**Affiliations:** 1 Energetic Materials Laboratory, Teaching and Research Unit of Energetic Processes, Ecole Militaire Polytechnique, BP 17, Bordj El-Bahri, Algiers 16046, Algeria; 2 Biorefining and Advanced Materials Research Center, Scotland’s Rural College (SRUC), Kings Buildings, Edinburgh EH9 3JG, UK; vijay.kumar@sruc.ac.uk; 3 Department of Mechanical Engineering, School of Engineering, Shiv Nadar University, Uttar Pradesh 201314, India

Since the emergence of nanotechnology in recent decades, the development and design of hybrid bio-nanomaterials has become an important field of research. Looking at the growing concern about the environment and sustainability, such nanomaterials find many applications in a wide range of domains that influence our society and our way of life [1]. The improvement in properties and the discovery of new functionalities are key goals that cannot be reached without a well-controlled and better understanding of the preparation, characterization, and manufacturing of new hybrid nanomaterials, which constitute the starting points of the design of specific and adequate systems [2,3]. Investigation of nanocellulose/nanocarbons hybrid materials has demonstrated both academic and technological importance and offered great research opportunities within cross-disciplinary areas.

Nanocellulose, which refers to the cellulose with a nanoscale dimension, encompasses cellulose nanocrystals, cellulose nanofibrils, and bacterial cellulose [4,5]. It displays outstanding features such as biocompatibility, eco-friendliness, renewability, interesting reinforcing potential, hydrogen-bonding capacity, low thermal expansion coefficient, dimensional stability, high elastic modulus, high specific surface area, and low density [6,7,8,9]. Various nanocellulose-based composites, which found application in different fields, have been developed during the last two decades. However, numerous challenges need to be addressed to achieve the full requirement of advanced materials [10,11,12,13,14]. Accordingly, several research activities continue to be performed by different research groups worldwide to develop the next generation of nanomaterials and fully explore the potential of nanocellulose.

Nanocarbon is another exciting type of potential nanomaterials, which has received tremendous attention since the discovery of fullerenes over thirty years ago [15,16,17,18]. They include carbon nanotubes, graphene and carbon dots. They exhibit outstanding thermal, electrical, optical and mechanical features, and they can be employed in a wide range of applications such as composites, microelectronics, biomedical, conductive films, sensors, adsorbents, catalysis, energy storage, and coating, among others.

Based on these premises, the present Special Issue in *Nanomaterials* aims to further contribute to the momentum of research and development in nanocellulose/nanocarbon hybrids, by featuring ten (10) original research articles as well as two (2) review articles, authored and reviewed by experts in the field. It targets a broad readership of materials scientists, chemists, physicists, and nanotechnologists, among others. Most of the research papers highlight theoretical concepts and practical approaches of interest for real-world applications related to nanocellulose and nanocarbons.

Some interesting research works dealing with nanocellulose-based materials have been published in the present Special Issue. Imtiaz et al. prepared wood-based cationic cellulose nanocrystals (CNCs) and assessed their cytotoxicity as potential immunomodulators [19]. They firstly transformed anionic CNCs to cationic CNCs with a rod-like shape through the grafting of cationic polymers containing pendant ^+^NMe_3_ and ^+^NH_3_. The authors proved that such an interesting cellulosic derivative, which exhibited very low toxicity, is considered as a good candidate as an immunomodulator and as vaccine nano-adjuvants. In another work, Han et al. investigated the physicochemical properties of cationic nanocellulose (cationic NC) and starch nanocomposites [20]. The authors prepared cationic NC from cationic-modified microcrystalline cellulose (MCC) using different approaches, i.e., acid hydrolysis, high-pressure homogenization, and high-intensity ultrasonication. They demonstrated that the nanocomposite prepared by cationic-NC produced by high-pressure homogenization and starch displayed a good dispersion, better thermal stability and the best water vapor barrier properties compared to other nanocomposites, which suggest that such nanocomposite can find potential application as a biodegradable food-packaging material. The study by Sharma et al. assessed the potential of carboxy cellulose nanofibers (CNF) extracted from untreated jute biomass using nitro-oxidation method as the reinforcing agent of natural rubber latex (NRL) [21]. It was revealed that the incorporation of CNF into NRL enhanced the mechanical characteristics even in the non-vulcanized state for which the optimal amount of CNF is around 0.2 wt.%. Another paper by Hai et al. describes the preparation and characterization of cellulose nanofibers and chitosan nanofiber blends, which are expected to be applied for active food packaging [22]. The obtained nanocomposite displayed a remarkable improvement in the mechanical features. The higher content of chitosan nanofibers exhibited higher antioxidant activity, better UV-light protection, increased water vapor transmission rate, and better mechanical properties. They claimed that such a nanocomposite could be useful for active food packaging application. The report by Lu et al. describes the preparation of a nanocellulose/metal-organic-framework-derived carbon-doped CuO/Fe_3_O_4_ nanocomposite via direct calcination [23]. Its catalytic efficiency through the reduction in 4-nitrophenol, methylene blue, and methyl orange has been demonstrated.

Similarly, interesting nanocarbon-based materials have been also reported in the present Special Issue. One research article focused on the reinforcement of high-molecular-weight, multimodal, high-density polyethylene by microwave-induced plasma graphene platelets using melt intercalation [24]. It was found that graphene platelets were homogenously distributed and dispersed within the polymer matrix through a strong interfacial bonding. The developed nanocomposites exhibited better thermal stability and interesting mechanical features. In another study, Sharma et al. developed a now-reduced graphene oxide incorporated gun tragacanth-cl-*N*, *N*-dimethylacrylamide hydrogel composite as a reusable adsorbent for Hg^2+^ and Cr^6+^ ions [25]. The authors optimized the experimental conditions of the synthesis based on the microwave-assisted method. It is proved that the adsorption effectiveness of 99% and 82% were obtained for Hg^2+^ and Cr^6+^ ions, respectively, confirming that the developed adsorbents are highly efficient and can be employed for environmental remediation applications. A pure nanocarbon hybrid based on multi-walled carbon nanotube and graphene nanoplatelet (GNP) for flexible strain sensors has been developed by Huang et al. [26]. The production of such hybrid was assisted with surfactant Triton X-100 and the sonication through a vacuum filtration process. The authors revealed that the increase in the content of GNP from 0 to 5% decreased the tensile strength of the hybrid film, whereas the electrical conductivity increased. It is also found that the increase of the GNP content leads to an increase in the strain sensitivity with good stability and repeatability.

Considered as a promising class of nanomaterials, the combination of nanocellulose and nanocarbon has received tremendous interest in recent years. This interest is promoted by the exceptional properties and outstanding synergetic effects that these powerful nanomaterials offer, which allow developing new opportunities and ways for the preparation and employment of novel materials in nanotechnology spanning from water treatment, energy and environment, optics and photonics, medical, biosensing and optoelectronics. For such specific nanohybrids, two review papers and two research articles have been published. The first review article compiles five years of recent research findings on new development on the emerging generation of cellulose nanocrystals/graphene-based nanomaterials [27]. The methodologies of their production, their properties and applications have been thoroughly discussed. It is demonstrated that such nano-hybrids exhibited prominent innovative features due to synergetic effects, which are unachievable by taking cellulose nanocrystals and graphene-based nanomaterials separately. The authors reported that the real use of such hybrids as the next generation of materials necessitates more efforts and further improvements in functionality and performance, in addition, the decrease in the production costs and environmental impacts. The second review by Bacakova et al. is dedicated to the application of nanocellulose (nanofibrils and nanocrystals)/nanocarbon (fullerenes, graphene, nanotubes and nanodiamonds) composites with a special focus on biotechnology and medicine [28]. Such promising hybrids displayed unique features such as high mechanical strength coupled with flexibility and stretchability, shape memory effect, photodynamic and photothermal activity, electrical conductivity, semiconductivity, thermal conductivity, tunable optical transparency, intrinsic fluorescence and luminescence, and high adsorption and filtration capacity. They can be used in several applications such as wound dressing, tissue engineering, electrical stimulation of damaged tissues, isolation of different biomolecules, drug delivery, among others. However, the authors claimed that the risk of their potential cytotoxicity and immulogenicity needs to be deeply investigated.

The first research paper dealing with nanocellulose/nanocarbon hybrids is studied by Yu et al. [29]. These authors prepared cellulose nanofibrils (CNFs)/carbon nanomaterial hybrid aerogels for adsorption removal of cationic and anionic organic dyes. They employed two classes of nanocarbons, i.e., carbon nanotubes (CNT) and graphene nanoplates (GnP), which are combined with different amounts of CNFs. They reported that the combination CNFs: GnP 3:1 provided the best performance toward day adsorption for which the pseudo-second-adored kinetic and monolayer Langmuir adsorption isotherm are followed. The authors claimed that such a hybrid could find promising application as an adsorption material for wastewater treatment. The last work presented in the Special Issue studied by Wang et al. describes the preparation and characterization of self-healable electro-conductive hydrogels based on core-shell structured nanocellulose/carbon nanotubes hybrids, expected to be used as flexible supercapacitors [30]. The developed hydrogel exhibited interesting mechanical features, high water-content, excellent flexibility, self-healing capability, higher electrical conductivity and specific capacity, rendering it a potential candidate for advanced personalized wearable electronic devices.

In summary, the present Special Issue advances not only our understanding of the emerging and significant role of nanocellulose and nanocarbons in several fields, but also of the challenges and future research directions needed to fully explore their outstanding features in practical ways. It is also expected that this Special Issue will encourage multidisciplinary research activities on hybrid bio-nanomaterials, extending the range of potential practical applications taking into account the scaling-up of the systems, the economic viability, the impact on the environment and human health as well as the long-term stability and recyclability.
